# Cancer-related Emergency Department Visits: Comparing Characteristics and Outcomes

**DOI:** 10.5811/westjem.2021.5.51118

**Published:** 2021-08-21

**Authors:** Rahul V. Nene, Jesse J. Brennan, Edward M. Castillo, Peter Tran, Renee Y. Hsia, Christopher J. Coyne

**Affiliations:** * University of California, San Diego, Department of Emergency Medicine, San Diego, California; † University of California, San Francisco, Department of Emergency Medicine, San Francisco, California; ‡ University of California, San Francisco, Institute for Health Policy Studies, San Francisco, California

## Abstract

**Introduction:**

There is increasing appreciation of the challenges of providing safe and appropriate care to cancer patients in the emergency department (ED). Our goal here was to assess which patient characteristics are associated with more frequent ED revisits.

**Methods:**

This was a retrospective cohort study of all ED visits in California during the 2016 calendar year using data from the California Office of Statewide Health Planning and Development. We defined revisits as a return visit to an ED within seven days of the index visit. For both index and return visits, we assessed various patient characteristics, including age, cancer type, medical comorbidities, and ED disposition.

**Results:**

Among 12.9 million ED visits, we identified 73,465 adult cancer patients comprising 103,523 visits that met our inclusion criteria. Cancer patients had a 7-day revisit rate of 17.9% vs 13.2% for non-cancer patients. Cancer patients had a higher rate of admission upon 7-day revisit (36.7% vs 15.6%). Patients with cancers of the small intestine, stomach, and pancreas had the highest rate of 7-day revisits (22–24%). Cancer patients younger than 65 had a higher 7-day revisit rate than the elderly (20.0% vs 16.2%).

**Conclusion:**

In a review of all cancer-related ED visits in the state of California, we found a variety of characteristics associated with a higher rate of 7-day ED revisits. Our goal in this study was to inform future research to identify interventions on the index visit that may improve patient outcomes.

## INTRODUCTION

Providing emergency care to cancer patients presents a unique set of challenges for the healthcare system. In 2015 the National Institutes of Health established a consortium to advance knowledge in this area, with one specific, highlighted aim as the collection of epidemiologic data.^1^ According to the 2015 National Hospital Ambulatory Medical Care Survey, cancer patients accounted for 3.4% of emergency department (ED) visits across all age groups.^2^ A recently published survey found that patients with cancer who present to the ED are more likely to be older, experience prolonged ED stays, and be admitted.^3^ However, there is a dearth of information regarding the epidemiology of those cancer patients who visit the ED and which factors lead to ED revisits. The short-term revisit rate is an increasingly analyzed quality metric as it is associated with worse outcomes, including morbidity and mortality.^4^–^7^ Furthermore, these early revisits may represent medical errors or failures in the healthcare delivery model and can help recognize targets for intervention.^8^

The ED operates as the primary healthcare access point for many of these cancer patients, and it is vital to understand how and why these patients present to the ED. Identification of the risk factors that lead to ED revisits can provide physicians who care for these patients with knowledge that might lead to improved patient outcomes. In this study our goal was to investigate which characteristics specific to cancer patients are associated with seven-day ED revisits, how this varies based on cancer type, and how age may affect ED revisits among the cancer population.

## METHODS

### Study Design

This multicenter, retrospective cohort study uses non-public data from January 1–December 31, 2016 from the California Office of Statewide Health Planning and Development (OSHPD). All non-military, licensed hospitals in the state are subject to mandatory reporting of utilization data in a standardized format to the OSHPD. The database includes 321 of the 334 hospitals (96.1%) in California with a licensed ED. We obtained approval for this study from the University of California at San Francisco institutional review board. This manuscript was developed and written in accordance with STROBE criteria.^9^

### Data Collection and Processing

We used data from two datasets for this study: the Patient Discharge Dataset and the Emergency Department Dataset. From the Patient Discharge Dataset we extracted data regarding patients who were admitted through the ED and then we merged that information with the Emergency Department Dataset to construct a complete ED utilization database. Data included the following: limited demographic characteristics; service date; hospital length of stay for admitted patients; discharge disposition; and primary diagnosis, plus up to 24 *International Classification of Disease 10th Revision Clinical Modification* (ICD-10-CM) diagnoses codes. Detailed information on these data sources is available elsewhere.^10^ We excluded patients < 18 years of age, patients without a valid patient identifier, and visits with a primary diagnosis of maternity.

We identified cancer patients with having at least one cancer-related ED visit in the study period by the primary or any secondary diagnosis using National Cancer Institute recommendations included the following ICD-10-CM codes: C00x to C26x; C30x to C41x; C43x to C58x; C60x to C96x; C7Ax to C7Bx; and D46x to D47x.^19^ Comorbidity was determined by using the primary and secondary ICD-10-CM codes to calculate a modified version of the Charlson Comorbidity Index (CMI) score,^11^ which were categorized as 0, 1, 2, and 3+. To compare CMI scores between cancer and non-cancer patients we excluded the categories of “Cancer” and “Metastatic Carcinoma.”

### Primary Data Analysis

We report the number and proportion of non-cancer and cancer patients who returned to the ED within seven days as broken down by disposition, including admission to the hospital, or discharge to home or a skilled nursing facility (including rehabilitation facilities and intermediate care facilities). The CMI scores for the overall population and adjusted scored for the cancer cohort are reported for those with a seven-day revisit. We calculated revisit rates for each cancer type and used bivariate logistic regression to calculate odds ratios (OR) for the increased likelihood of seven-day return visit for each cancer type. We further compared non-cancer and cancer patients by disposition from the ED after a revisit within seven days by age (< 65 vs 65 and older). All data analyses were completed at the visit level. We conducted statistical analyses using the SPSS Statistics 25.0 software package (IBM Corporation, Armonk, NY). Given the very large sample size and the associated power, we omitted *P*-values in our study results given that essentially all comparisons would appear significant.

**Population Health Research Capsule**

What do we already know about this issue?

*Cancer patients account for 3.4% of emergency department (ED) visits. They are more likely to be older, experience prolonged ED stays, and more likely to be admitted*.

What was the research question?

*Which characteristics specific to cancer patients are associated with 7-day ED revisits?*

What was the major finding of the study?

*Cancer patients younger than 65 and those with gastrointestinal cancers had a higher 7-day ED revisit rate*.

How does this improve population health?

*Awareness of cancer-patient characteristics associated with more frequent ED revisits may help identify interventions on the index visit to improve patient outcomes*.

## RESULTS

### ED Revisits

There were 12.9 million ED visits during the 2016 calendar year for initial ED visits and subsequent revisits. A total of 73,465 adult cancer patients comprised 103,523 visits that met our inclusion criteria. Approximately 5% of patients had invalid patient identifiers and were excluded from this analysis. Among all adult cancer visits, 17.9% resulted in a seven-day ED revisit (18,491 subsequent visits), higher than the 13.2% revisit rate we found for non-cancer visits. Table 1 shows the demographics of cancer patients vs non-cancer patients who had a seven-day ED revisit. On average, compared to non-cancer patients, cancer patients who returned to the ED were more likely to be older, White, and have insurance through Medicare. These demographic differences were also generally reflected among patients who did not have a seven-day revisit. When patients returned to the ED, the primary discharge diagnosis changed approximately 75% of the time, with that rate of adjustment slightly higher in cancer patients compared to their non-cancer cohort (82.2% vs 74.6%). For revisits, a higher proportion of cancer patients returned to the same ED than non-cancer patients (77.3% vs 67.8%). The five most common ED diagnoses among cancer patient revisits, in descending order, were sepsis (5.7%); abdominal pain (4.9%); other pain (3.2%); chest pain (2.3%); and nausea/vomiting (2.3%) (Supplemental Table 1). Compared to their non-cancer cohort, cancer patients were admitted much more often upon seven-day revisit (36.7% vs 15.6%) (Supplemental Table 2).

The most prominent comorbidities for cancer patients at the revisit encounter included diabetes mellitus (23.9%); chronic pulmonary disease (14.9%); and vasculopathy (15.5%) among modified CMI categories (Supplemental Table 3). We also analyzed whether patients with multiple comorbidities accounted for more revisits (Table 2). Two-thirds of non-cancer patients with a seven-day revisit had no medical comorbidities, while this proportion decreased to one-half for cancer patients. Even corrected for presence of cancer, the CMI scores were still higher in the cancer population, emphasizing the fact that cancer patients who returned to the ED potentially had multiple factors contributing to an ED revisit compared to the non-cancer cohort.

### Specific Cancer Types with Higher Revisit Rates

Cancers accounting for the most ED revisits included lung cancer, breast cancer, prostate cancer, and non-Hodgkin’s lymphoma, consistent with the high prevalence of these cancers in the community (Table 3). Compared to the revisit rate for all cancer patients, cancers of the gastrointestinal system had the highest revisit rates, including cancers of the small intestine (OR 1.48, confidence interval [CI], 1.02, 2.15); liver (OR 1.49, CI, 1.37, 1.61); and pancreas (OR 1.43, CI, 1.32, 1.55). In contrast, the more common breast and prostate cancers had significantly lower revisit rates (OR 0.72, CI, 0.68, 0.76 and OR 0.90, CI, 0.85, 0.95, respectively). Cancers traditionally considered to be higher risk, such as brain cancer, ovarian cancer, and melanoma^12^ did not have significantly increased or decreased rates of revisit relative to the overall revisit rate.

We also assessed outcomes for the subset of patients who had secondary metastases (26,890 patients accounting for 44,075 visits). Among this group we observed a higher rate of seven-day ED revisits (21.6% vs 17.9%), a higher rate of admission on the second ED visit (41.4% vs 36.7%), and a higher mortality during that admission (9.6% vs 8.4%), compared to cancer patients without metastases. The top five primary cancers that had the highest revisit rates when complicated by metastasis were as follows: myeloid leukemia (47.8%); testicular cancer (35.1%); Hodgkin’s lymphoma (32.8%); cervical cancer (29%); and stomach cancer (28.8%) (Supplemental Table 4).

### Variation in Outcomes Between Younger and Older Cancer Patients

Of the 103,523 visits that met our inclusion criteria, 56% (57,955) were by patients ≥ 65 years of age. The seven-day revisit rate was lower for elderly cancer patients at 16.2% vs 20.0% in those younger than 65. However, during that seven-day ED revisit, elderly cancer patients had a higher rate of admission diagnosis than younger patients (6.8%) (Supplemental Table 6), while septicemia was the most common ED diagnosis in the elderly (6.7%) (Supplemental Table 7). The most common diagnosis resulting in admission for both age groups was septicemia (12.8% in younger patients and 16.4% in elderly patients) (Supplemental Tables 8 and 9). Furthermore, elderly cancer patients were more likely to expire during that admission (8.8% vs 8.0%). When discharged from either the ED or after an admission, the elderly were placed in a skilled nursing facility or discharged with home health services more often. Among cancer patients under 65, gastrointestinal cancers still accounted for the highest revisit rates, though were even higher at 25–28%. Among the elderly, cancers of the gastrointestinal system also accounted for the greatest rates of revisits (~17–20%). All cancers had higher revisit rates in the young, except for hematologic malignancies, which appeared to have equal rates in the elderly.

## DISCUSSION

A recently published national survey identified factors that lead to adult cancer patient ED visits and subsequent hospital admission.^14^ Similar to that study, we found a much higher rate of admission for cancer patients compared to non-cancer patients. We also found sepsis/infection was the most common reason for admission. However, in contrast to that prior study, which focused only on index visits, we chose to study which factors account for early ED revisits. We found that cancer patients have a significantly higher rate of \seven-day revisit compared to a non-cancer cohort and are twice as likely to be admitted upon that revisit. Unsurprisingly, the presence of metastatic disease was the most prominent feature among ED revisits. Other medical comorbidities also contributed significantly to the rates of ED revisits, including chronic pulmonary disease, poorly controlled diabetes, and renal disease. These data suggest that while the patient’s active cancer may be the most prominent factor leading to their ED visit, it is also important to address their additional medical diseases, which no doubt contribute to the patient’s morbidity and mortality.

Breast, prostate, and lung cancers, being the most prevalent cancers in the population, also contributed to the greatest number of ED revisits. However, it was certain rarer cancers that had the highest percentage of revisits, particularly cancers of the small intestine, stomach, and pancreas. This likely reflects the increased morbidity and mortality of these cancers, and the more vigorous medical and surgical therapies they require, factors that providers should keep in mind on the index visit. Hematologic malignancies also contributed to high rates of revisits, especially acute myeloid leukemia. Again, this may be due to the aggressive nature of these cancers and their treatments, or due to the immunosuppression that leaves these patients particularly vulnerable to infection and other comorbidities.

It is estimated that by 2030, 70% of all cancers will occur among patients aged > 65.^13^ Interestingly, it appears that younger cancer patients bounced back more frequently to the ED. Possible explanations include that ED providers feel more confident sending young patients home, or younger patients could have more aggressive cancers and receive more intensive chemotherapy, phenomena that are well characterized for breast and colorectal cancers.^15^–^17^ Although elderly patients tend to return to the ED less often, those that do require a repeat visit appear to have a higher admission rate and an increased mortality during that admission. Sepsis was the most likely reason for admission upon revisit for both the young and elderly cohorts. Perhaps future studies can determine whether obtaining an expanded infectious workup may be warranted for these patients on index visits, particularly when they present with vague, nonspecific symptoms.

We have identified several factors that are associated with higher rates of ED revisits for cancer patients and, in particular, we have highlighted factors that differentiate elderly cancer patients from a younger cohort. Emergency physicians, oncologists, primary care physicians, and all providers involved in the care of these patients should incorporate this knowledge into their disposition decisions and pay careful attention to those characteristics that place patients at the highest risk for repeat visit. For example, oncologists or primary care physicians could consider providing more detailed education regarding expected symptoms or even consider alternative care models where patients could bypass the ED. Emergency providers, for example, could consider keeping patients with a higher risk of deterioration for observation in the ED. The use of ED observational units has been particularly effective at avoiding unnecessary admissions in the treatment of chronic heart failure and atrial fibrillation.^18^–^20^ There is also an increasing utilization of observational units in the emergency care of geriatric patients, where a patient’s condition is allowed to evolve over the course of several hours, at which point a more informed decision can be made about admitting the patient or discharging with close follow-up.^21^ Alternatively, special efforts can be made to establish home health services for these patients and to coordinate urgent outpatient follow-up with their oncologists or primary care providers. These strategies have been proven to decrease ED revisits, particularly with geriatric care,^22^–^24^ while there is growing data on the effectiveness of these programs for cancer patients.^25^

## LIMITATIONS

The data accessed from a statewide database (OSHPD) had notable limitations including a small proportion of invalid patient identifiers (5%), the absence of federal healthcare facilities, and a lack of potentially important patient and visit characteristics including urgency, access to primary care, and cost, which would have been helpful to this study. Neither did we have access to visit-specific data, such as patient vitals, laboratory or imaging results, or provider rationale for admission vs discharge. And because these data were limited to facilities within California our findings may not be generalizable to other patient populations.

This study was also limited to data captured by ED databases, thereby resulting in censoring whether patients died at home prior to a seven-day revisit. This censoring may have affected revisit rates among those with more aggressive/advanced cancers and among the elderly. All revisit rates were calculated at the level of visits, thereby accounting for patients who had multiple ED visits during the study period. This potentially raises the issue of data being skewed by a small number of “super users” who have frequent revisits. We looked at this briefly at the overall cancer population level and found that of the 73,465 cancer patients who visited the ED in 2016, 13,977 had at least one seven-day revisit, for a revisit rate of 19.0%, which is slightly higher than (although similar to) the overall revisit rate of 17.9%. While generally reassuring, it is possible that there was skewing by frequent users in our subgroup analyses, such as revisit rates for the rarer gastrointestinal cancers.

## CONCLUSION

We have conducted what is to our knowledge the first comprehensive analysis assessing ED revisits for cancer patients, and potential factors associated with revisits that occurred within seven days of the index visit. We hope these findings will serve as a steppingstone toward further studies that will help identify how we can better care for this high-risk population.

## Supplementary Information





## Figures and Tables

**Table 1:** Demographics of patients on initial emergency department (ED visit vs seven-day ED revisits).

	Initial ED Visit				7-Day ED Revisit			
		
	Non-Cancer Patient Encounters		Cancer Patient Encounters		Non-Cancer Patient Encounters		Cancer Patient Encounters	
				
	N	%	N	%	N	%	N	%
Gender								
Male	1,959,633	44.4	28,380	47.7	486,269	49.5	7,138	51.1
Female	2,452,122	55.6	31,106	52.3	496,935	50.5	6,839	48.9
Age								
18 – 44	2,101,554	47.7	5,036	8.5	464,031	47.2	1,411	10.1
45 – 65	1,358,362	30.8	19,371	32.6	339,465	34.6	4,922	35.2
65 – 84	760,255	17.2	27,648	46.5	140,844	14.3	6,294	45.0
85+	191,740	4.3	7,433	12.5	38,885	4.0	1,350	9.7
Race/Ethnicity								
Hispanic	1,394,811	31.6	11,491	19.3	278,976	28.4	2,866	20.5
NH White	1,973,305	44.7	35,104	59.0	456,586	46.4	7,928	56.7
NH Black	462,675	10.5	5,402	9.1	160,926	16.4	1,456	10.4
NH Asian	319,354	7.2	5,072	8.5	41,287	4.2	1,185	8.5
Payor Status								
Private	1,653,766	37.5	15,733	26.4	205,260	20.9	3,277	23.4
Medicare	1,025,847	23.3	34,423	57.9	254,138	25.8	7,706	55.1
Medi-Cal	1,367,867	31.0	8,245	13.9	448,134	45.6	2,744	19.6
Self-pay/Indigent	364,431	8.3	1,087	1.8	75,693	7.7	250	1.8

*ED*, emergency department; *NH*, Non-Hispanic.

**Table 2:** Comorbidity index score category associated with seven-day emergency department revisits.

CMI Score	Non-Cancer Patient Encounters		Cancer Patient Encounters	
	
	N	%	N	%
0	668,472	68.0	9,302	50.3
1	183,437	18.7	4,195	22.7
2	58,268	5.9	2,059	11.1
3+	73,048	7.4	2,935	15.9
Total	983,225	100.0	18,491	100.0

*CMI*, comorbidity index.

**Table 3:** Seven-day revisit rate by cancer type.

	Index Visits	7-Day Revisits	7-Day Revisit Rate	Bivariate OR (95% CI)
Breast (Female)	10,933	1,514	13.8%	0.74 (0.70, 0.79)
Lung	9,418	1,805	19.2%	1.10 (1.04, 1.16)
Prostate	9,405	1,549	16.5%	0.82 (0.78, 0.87)
Myelodysplastic syndrome	6,879	1,042	15.1%	0.81 (0.76, 0.87)
Non-Hodgkin’s lymphoma	5,711	982	17.2%	0.95 (0.89, 1.02)
Colon	4,818	912	18.9%	1.08 (1.00, 1.16)
Multiple myeloma	4,409	697	15.8%	0.86 (0.79, 0.93)
Lymphoid leukemia	3,757	613	16.3%	0.89 (0.82, 0.98)
Liver	3,480	840	24.1%	1.49 (1.37, 1.61)
Pancreas	3,409	800	23.5%	1.43 (1.32, 1.55)
Ovarian	2,623	475	18.1%	1.09 (0.98, 1.20)
Bladder	2,558	473	18.5%	1.04 (0.94, 1.16)
Lip, oral cavity, and pharynx	2,113	463	21.9%	1.30 (1.17, 1.44)
Kidney	1,981	351	17.7%	0.99 (0.88, 1.11)
Brain	1,968	321	16.3%	0.89 (0.79, 1.01)
Uterine	1,948	351	18.0%	1.08 (0.96, 1.21)
Myeloid and monocytic leukemia	1,917	413	21.5%	1.27 (1.14, 1.42)
Stomach	1,706	391	22.9%	1.38 (1.23, 1.54)
Esophagus	1,291	276	21.4%	1.25 (1.10, 1.43)
Cervical	1,249	277	22.2%	1.40 (1.23, 1.61)
Melanoma	1,017	166	16.3%	0.90 (0.76, 1.06)
Thyroid	1,000	140	14.0%	0.75 (0.62, 0.89)
Hodgkin’s lymphoma	888	141	15.9%	0.87 (0.72, 1.04)
Bones and joints	600	108	18.0%	1.01 (0.82, 1.24)
Neuroendocrine tumors	434	79	18.2%	1.02 (0.80, 1.31)
Larynx	341	76	22.3%	1.32 (1.02, 1.71)
Anus	277	68	24.5%	1.50 (1.14, 1.97)
Kaposi sarcoma	153	32	20.9%	1.22 (0.82, 1.80)
Small Intestine	152	37	24.3%	1.48 (1.02, 2.15)
Eye and orbit	91	19	20.9%	1.21 (0.73, 2.01)

*OR*, odds-ratio; *CI*, confidence interval.

## References

[1] Brown J, Grudzen C, Kyriacou DN (2016). The emergency care of patients with cancer: setting the research agenda. Ann Emerg Med.

[2] Rui P, Kang K (2015). National Hospital Ambulatory Medical Care Survey: 2015. Emergency Department Summary Tables.

[3] Hsu J, Donnelly JP, Moore JX (2018). National characteristics of emergency department visits by patients with cancer in the United States. Am J Emerg Med.

[4] Schull MJ, Guttmann A, Leaver CA (2011). Prioritizing performance measurement for emergency department care: consensus on evidence-based quality of care indicators. CJEM.

[5] Pierce JM, Kellerman AL, Oster C (1990). “Bounces”: an analysis of short-term return visits to a public hospital emergency department. Ann Emerg Med.

[6] Lyons TW, Olson KL, Palmer NP (2017). Patients visiting multiple emergency departments: patterns, costs, and risk factors. Acad Emerg Med.

[7] de Gelder J, Lucke JA, de Groot B (2018). Predictors and outcomes of revisits in older adults discharged from the emergency department. J Am Geriatr Soc.

[8] Gabayan GZ, Asch SM, Hsia RY (2013). Factors associated with short-term bounce-back admissions after emergency department discharge. Ann Emerg Med.

[9] von Elm E, Altman DG, Egger M (2007). The Strengthening the Reporting of Observational Studies in Epidemiology (STROBE) statement: guidelines for reporting observational studies. Ann Intern Med.

[10] California Office of Statewide Health Planning and Development MIRCal - Medical Information Reporting for California.

[11] Quan H, Sundararajan V, Halfon P (2005). Coding algorithms for defining comorbidities in ICD-9-CM and ICD-10 administrative data. Med Care.

[12] Budczies J, von Winterfeld M, Klauschen F (2015). The landscape of metastatic progression patterns across major human cancers. Oncotarget.

[13] White MC, Holman DM, Boehm JE (2014). Age and cancer risk: a potentially modifiable relationship. Am J Prev Med.

[14] Rivera DR, Gallicchio L, Brown J (2017). Trends in adult cancer-related emergency department utilization: an analysis of data from the Nationwide Emergency Department Sample. JAMA Oncol.

[15] Abdelsattar ZM, Wong SL, Regenbogen SE (2016). Colorectal cancer outcomes and treatment patterns in patients too young for average-risk screening. Cancer.

[16] Fredholm H, Eaker S, Frisell J (2009). Breast cancer in young women: poor survival despite intensive treatment. PLoS One.

[17] Anders CK, Johnson R, Litton J (2009). Breast cancer before age 40 years. Semin Oncol.

[18] Bellew SD, Bremer ML, Kopecky SL (2016). Impact of an emergency department observation unit management algorithm for atrial fibrillation. J Am Heart Assoc.

[19] Ross MA, Aurora T, Graff L (2012). State of the art: emergency department observation units. Crit Pathw Cardiol.

[20] Blecker S, Ladapo JA, Doran KM (2014). Emergency department visits for heart failure and subsequent hospitalization or observation unit admission. Am Heart J.

[21] Moseley MG, Hawley MP, Caterino JM (2013). Emergency department observation units and the older patient. Clin Geriatr Med.

[22] Pines JM, Keyes V, Van Hasselt M (2015). Emergency department and inpatient hospital use by Medicare beneficiaries in patient-centered medical homes. Ann Emerg Med.

[23] Nyweide DJ, Bynum JPW (2017). Relationship between continuity of ambulatory care and risk of emergency department episodes among older adults. Ann Emerg Med.

[24] Woods LW, Snow SW (2013). The impact of telehealth monitoring on acute care hospitalization rates and emergency department visit rates for patients using home health skilled nursing care. Home Heal Nurse.

[25] Colligan EM, Ewald E, Keating NL (2017). Two innovative cancer care programs have potential to reduce utilization and spending. Med Care.

