# Supplementary material for: Cancer-related Emergency Department Visits: Comparing Characteristics and Outcomes
Source: West J Emerg Med. 2021 Aug 21;22(5):1117–23. doi: 10.5811/westjem.2021.5.51118 (PMC8463053; doi:10.5811/westjem.2021.5.51118)
Supplement: Supplementary file 1 [file wjem-22-1117-s001.docx]

**Supplemental Table 1.** Most common diagnoses for cancer patients upon seven-day revisit.

| **ICD-10 Diagnosis** | **N** | **%** |
| --- | --- | --- |
| Sepsis | 1058 | 5.7 |
| Abdominal pain | 899 | 4.9 |
| Other pain | 592 | 3.2 |
| Chest or throat pain | 427 | 2.3 |
| Nausea and vomiting | 425 | 2.3 |
| Urinary tract infection | 344 | 1.9 |
| Back pain | 332 | 1.8 |
| Pneumonia | 299 | 1.6 |
| Metastatic malignancy to other sites | 286 | 1.5 |
| Complications of genitourinary prosthetic devices, implants, and grafts | 264 | 1.4 |
| Malaise and fatigue | 258 | 1.4 |
| Secondary malignant neoplasm of respiratory and digestive organs | 257 | 1.4 |
| Shortness of breath | 256 | 1.4 |
| Dehydration | 254 | 1.4 |
| Other functional intestinal disorders | 246 | 1.3 |
| Lung cancer | 243 | 1.3 |
| Paralytic ileus and intestinal obstruction without hernia | 207 | 1.1 |
| Anemia | 201 | 1.1 |
| Cellulitis | 198 | 1.1 |
| Ascites | 195 | 1.1 |
| Urinary retention | 173 | 0.9 |
| Chronic obstructive pulmonary disease | 163 | 0.9 |
| Acute renal failure | 160 | 0.9 |
| Headache | 159 | 0.9 |
| Fever of unknown origin | 155 | 0.8 |
| Agranulocytosis | 151 | 0.8 |
| Heart failure | 142 | 0.8 |
| Noninfective gastroenteritis and colitis | 141 | 0.8 |
| Syncope and collapse | 141 | 0.8 |
| Other disorders of fluid, electrolyte, and acid-base balance | 139 | 0.8 |
| Other soft tissue disorders, not elsewhere classified | 139 | 0.8 |
| Other diseases of digestive system | 137 | 0.7 |
| Other symptoms and signs involving the digestive system and abdomen | 136 | 0.7 |
| Pleural effusion | 134 | 0.7 |
| Hematuria | 133 | 0.7 |
| Non-insulin-dependent diabetes mellitus | 126 | 0.7 |
| Respiratory failure | 125 | 0.7 |
| Hemoptysis | 125 | 0.7 |
| Atrial fibrillation and flutter | 124 | 0.7 |

**Supplemental Table 2.** Emergency department (ED) disposition after seven-day ED revisit.

|  | **Non-Cancer Patient Encounters** | | **Cancer Patient Encounters** | |
| --- | --- | --- | --- | --- |
| **Discharge Disposition** | **N** | **%** | **N** | **%** |
| Admitted/transferred to acute care facility | 152,933 | 15.6 | 6,779 | 36.7 |
| Discharged to home | 784,691 | 79.7 | 10,924 | 59.2 |
| Discharged with home health service | 2,770 | 0.3 | 174 | 0.9 |
| Discharged to skilled nursing facility | 6,394 | 0.7 | 225 | 1.2 |
| Left against medical advice | 35,340 | 3.6 | 285 | 1.5 |
| Expired | 609 | 0.1 | 100 | 0.5 |

**Supplemental Table 3.** Most common medical comorbidities for cancer patient with seven-day revisits.

| **Disease Diagnosis** | **N** | **%** |
| --- | --- | --- |
| Metastatic carcinoma | 5,329 | 28.8% |
| Diabetes w/o complications | 3,307 | 17.9% |
| Chronic pulmonary disease | 2,754 | 14.9% |
| Renal disease | 2,443 | 13.2% |
| Peripheral vascular disease | 1,578 | 8.5% |
| Congestive heart failure | 1,490 | 8.1% |
| Diabetes w/ complications | 1,103 | 6.0% |
| Mild liver disease | 795 | 4.3% |
| Myocardial infarction | 770 | 4.2% |
| Cerebrovascular disease | 524 | 2.8% |
| Dementia | 508 | 2.7% |
| Mod or severe liver disease | 326 | 1.8% |
| Rheumatic disease | 276 | 1.5% |
| Paraplegia and hemiplegia | 171 | 0.9% |
| Peptic ulcer disease | 124 | 0.7% |
| AIDS/HIV | 69 | 0.4% |

*Mod*, moderate; *AIDS/HIV*, acquired immunodeficiency syndrome/human immunodefiency virus.

**Supplemental Table 4.** Primary metastatic cancers with the highest seven-day revisit rates.

|  | **Index Visits** | **7-Day Revisits** | **7-Day Revisit Rate** |  |
| --- | --- | --- | --- | --- |
|  |  |  |  |  |
| Myeloid leukemia | 69 | 33 | 47.8% |  |
| Testicular | 268 | 94 | 35.1% |  |
| Hodgkin’s lymphoma | 61 | 20 | 32.8% |  |
| Cervical | 572 | 166 | 29.0% |  |
| Stomach | 1047 | 302 | 28.8% |  |
| Pancreas | 2160 | 597 | 27.6% |  |
| Liver | 1346 | 366 | 27.2% |  |
| Small intestine | 108 | 29 | 26.8% |  |
| Non-Hodgkin’s lymphoma | 556 | 149 | 26.8% |  |
| Lymphoid leukemia | 152 | 40 | 26.3% |  |
| Other endocrine system | 51 | 13 | 25.5% |  |
| Multiple myeloma | 494 | 122 | 24.7% |  |
| Anus | 78 | 19 | 24.4% |  |
| Esophagus | 689 | 164 | 23.8% |  |
| Brain and nervous system | 287 | 68 | 23.7% |  |
| Eye and orbit | 38 | 9 | 23.7% |  |
| Bladder | 733 | 171 | 23.3% |  |
| Other digestive organ | 262 | 61 | 23.3% |  |
| Lip, oral cavity, and pharynx | 894 | 208 | 23.3% |  |
| Uterine | 917 | 209 | 22.8% |  |
| Bones and joints | 288 | 65 | 22.6% |  |
| Colon | 2927 | 649 | 22.2% |  |
| Soft tissue | 452 | 98 | 21.7% |  |
| Larynx | 112 | 24 | 21.4% |  |
| Lung | 5582 | 1186 | 21.2% |  |
| Thyroid | 339 | 72 | 21.2% |  |
| Melanoma | 505 | 104 | 20.6% |  |
| Prostate | 3769 | 773 | 20.5% |  |
| Kidney | 1035 | 211 | 20.4% |  |
| Ovarian | 1683 | 339 | 20.1% |  |
| Rectum | 928 | 186 | 20.0% |  |
| Neuroendocrine tumors | 305 | 61 | 20.0% |  |
| Breast (Female) | 4789 | 897 | 18.7% |  |
| Breast (Male) | 43 | 1 | 2.3% |  |
| Mycosis fungoides | 1 | 0 | 0.0% |  |

| **Supplemental Table 5.** Outcomes difference between younger and older cancer patients. | | | | | | |
| --- | --- | --- | --- | --- | --- | --- |
|  | **Emergency Department Disposition after 7-Day ED Revisit** | | | | | |
|  | **Discharged to Home** | | **Admitted** | | **Expired** | |
|  | **N** | **%** | **N** | **%** | **N** | **%** |
| **Non-Cancer Patient Encounters < 65** | 662,773 | 82.5 | 104,320 | 13.0 | 215 | < 0.1 |
| **Cancer Patient Encounters < 65** | 5,629 | 61.7 | 3,170 | 34.7 | 37 | 0.4 |
| **Non-Cancer Patient Encounters ≥ 65** | 121,918 | 67.8 | 48,613 | 27.0 | 394 | 0.2 |
| **Cancer Patient Encounters≥ 65** | 5,295 | 56.5 | 3,609 | 38.5 | 63 | 0.7 |

**Supplemental Table 6.** Emergency department diagnosis for seven-day revisits among cancer patients < 65 years old.

| **ICD-10 Diagnosis Category** | **N** | **%** |
| --- | --- | --- |
| Abdominal and pelvic pain | 624 | 6.8 |
| Pain, not elsewhere classified | 441 | 4.8 |
| Sepsis | 428 | 4.7 |
| Nausea and vomiting | 282 | 3.1 |
| Chest and throat pain | 250 | 2.7 |
| Back pain | 189 | 2.1 |
| Secondary malignant neoplasm of other sites | 166 | 1.8 |
| Secondary malignant neoplasm of respiratory and digestive organs | 141 | 1.5 |
| Other disorders of urinary system | 131 | 1.4 |
| Ascites | 132 | 1.4 |
| Headache | 123 | 1.3 |
| Pneumonia | 113 | 1.2 |
| Other functional intestinal disorders | 107 | 1.2 |
| Abnormalities of breathing | 108 | 1.2 |
| Malignant neoplasm of bronchus and lung | 97 | 1.1 |
| Dehydration | 102 | 1.1 |
| Paralytic ileus and intestinal obstruction without hernia | 98 | 1.1 |
| Other noninfective gastroenteritis and colitis | 88 | 1.0 |
| Cellulitis | 88 | 1.0 |
| Other soft tissue disorders, not elsewhere classified | 91 | 1.0 |
| Anemia | 86 | 0.9 |
| Agranulocytosis | 80 | 0.9 |
| Use of alcohol | 81 | 0.9 |
| Fever of unknown origin | 85 | 0.9 |
| Malaise and fatigue | 83 | 0.9 |
| Malignant neoplasm of breast | 74 | 0.8 |
| Other symptoms and signs involving the digestive system and abdomen | 73 | 0.8 |
| Other anxiety disorders | 63 | 0.7 |
| Chronic obstructive pulmonary disease | 68 | 0.7 |
| Other diseases of digestive system | 63 | 0.7 |
| Malignant neoplasm of colon | 54 | 0.6 |
| Malignant neoplasm of pancreas | 53 | 0.6 |
| Other disorders of fluid, electrolyte and acid-base balance | 57 | 0.6 |
| Epilepsy | 59 | 0.6 |
| Venous embolism and venous thrombosis | 57 | 0.6 |
| Pleural effusion, not elsewhere classified | 58 | 0.6 |
| Complications of artificial openings of the digestive system | 53 | 0.6 |

*ICD-10*, International Classification of Diseases, 10^th^ Modification.

**Supplemental Table 7.** Emergency department diagnosis for seven-day revisits among cancer patients > 65 years old.

| **ICD-10 Diagnosis Category** | **N** | **%** |
| --- | --- | --- |
| Sepsis | 630 | 6.7 |
| Abdominal and pelvic pain | 275 | 2.9 |
| Complications of genitourinary prosthetic devices, implants and grafts | 221 | 2.4 |
| Other disorders of urinary system | 213 | 2.3 |
| Pneumonia | 186 | 2.0 |
| Chest and throat pain | 177 | 1.9 |
| Malaise and fatigue | 175 | 1.9 |
| Malignant neoplasm of bronchus and lung | 146 | 1.6 |
| Dehydration | 152 | 1.6 |
| Pain, not elsewhere classified | 151 | 1.6 |
| Shortness of breath | 148 | 1.6 |
| Other functional intestinal disorders | 139 | 1.5 |
| Back pain | 143 | 1.5 |
| Nausea and vomiting | 143 | 1.5 |
| Secondary malignant neoplasm of other sites | 120 | 1.3 |
| Urinary retention | 124 | 1.3 |
| Secondary malignant neoplasm of respiratory and digestive organs | 116 | 1.2 |
| Anemia | 115 | 1.2 |
| Heart failure | 117 | 1.2 |
| Paralytic ileus and intestinal obstruction without hernia | 109 | 1.2 |
| Cellulitis | 110 | 1.2 |
| Acute renal failure | 113 | 1.2 |
| Atrial fibrillation and flutter | 105 | 1.1 |
| Hematuria | 106 | 1.1 |
| Syncope and collapse | 104 | 1.1 |
| Non-insulin-dependent diabetes mellitus | 89 | 1.0 |
| Chronic obstructive pulmonary disease | 95 | 1.0 |
| Other disorders of fluid, electrolyte, and acid-base balance | 82 | 0.9 |
| Respiratory failure | 82 | 0.9 |
| Hemoptysis | 86 | 0.9 |
| Altered mental status | 88 | 0.9 |
| Agranulocytosis | 71 | 0.8 |
| Pleural effusion | 76 | 0.8 |
| Other diseases of digestive system | 74 | 0.8 |
| Malignant neoplasm of pancreas | 63 | 0.7 |
| Ascites | 63 | 0.7 |
| Other symptoms and signs involving the digestive system and abdomen | 63 | 0.7 |
| Fever of unknown origin | 70 | 0.7 |
| Purpura and other hemorrhagic conditions | 53 | 0.6 |
| Other noninfective gastroenteritis and colitis | 53 | 0.6 |
| Other diseases of anus and rectum | 55 | 0.6 |
| Complications of artificial openings of the digestive system | 58 | 0.6 |
| Other joint disorders, not elsewhere classified | 58 | 0.6 |
| Dizziness | 53 | 0.6 |
| Attention to artificial openings | 58 | 0.6 |
| Fitting and adjustment of other devices | 56 | 0.6 |

*ICD-10*, International Classification of Diseases, 10^th^ Modification.

**Supplemental Table 8.** Primary diagnosis associated with admission upon seven-day revisit among cancer patients < 65 years old.

| **ICD-10 Diagnosis Category** | **N** | **%** |
| --- | --- | --- |
| Sepsis | 406 | 12.8 |
| Pain, not elsewhere classified | 164 | 5.2 |
| Secondary malignant neoplasm of other sites | 123 | 3.9 |
| Secondary malignant neoplasm of respiratory and digestive organs | 118 | 3.7 |
| Paralytic ileus and intestinal obstruction without hernia | 77 | 2.4 |
| Pneumonia | 74 | 2.3 |
| Agranulocytosis | 60 | 1.9 |
| Malignant neoplasm of bronchus and lung | 52 | 1.6 |
| Other noninfective gastroenteritis and colitis | 49 | 1.5 |
| Nausea and vomiting | 47 | 1.5 |
| Respiratory failure, not elsewhere classified | 37 | 1.2 |
| Other diseases of digestive system | 38 | 1.2 |
| Acute renal failure | 38 | 1.2 |
| Dehydration | 34 | 1.1 |
| Pulmonary embolism | 36 | 1.1 |
| Malignant neoplasm of stomach | 32 | 1.0 |
| Malignant neoplasm of colon | 32 | 1.0 |
| Malignant neoplasm of pancreas | 28 | 0.9 |
| Cellulitis | 27 | 0.9 |
| Abdominal and pelvic pain | 29 | 0.9 |
| Malignant neoplasm of breast | 26 | 0.8 |
| Malignant neoplasm of brain | 25 | 0.8 |
| Other venous embolism and venous thrombosis | 25 | 0.8 |
| Chest and throat pain | 25 | 0.8 |
| Complications following infusion or transfusion | 26 | 0.8 |
| Malignant neoplasms of liver | 21 | 0.7 |
| Diffuse non-Hodgkin’s lymphoma | 23 | 0.7 |
| Aplastic anemia | 21 | 0.7 |
| Other disorders of fluid, electrolyte, and acid-base balance | 23 | 0.7 |
| Chronic obstructive pulmonary disease | 22 | 0.7 |
| Disorders of continuity of bone | 21 | 0.7 |
| Other disorders of urinary system | 23 | 0.7 |
| Complications of procedures, not elsewhere classified | 21 | 0.7 |
| Malignant neoplasm of ovary | 19 | 0.6 |
| Multiple myeloma and malignant plasma cell neoplasms | 18 | 0.6 |
| Epilepsy | 19 | 0.6 |
| Hepatic failure | 20 | 0.6 |

*ICD-10*, International Classification of Diseases, 10^th^ Modification.

**Supplemental Table 9.** Primary diagnosis associated with admission upon seven-day revisit among cancer patients > 65 years old.

| Sepsis | 593 | 16.4 |
| --- | --- | --- |
| Pneumonia | 111 | 3.1 |
| Secondary malignant neoplasm of other sites | 95 | 2.6 |
| Acute renal failure | 93 | 2.6 |
| Paralytic ileus and intestinal obstruction without hernia | 87 | 2.4 |
| Secondary malignant neoplasm of respiratory and digestive organs | 83 | 2.3 |
| Malignant neoplasm of bronchus and lung | 76 | 2.1 |
| Heart failure | 73 | 2.0 |
| Respiratory failure, not elsewhere classified | 60 | 1.7 |
| Other disorders of urinary system | 63 | 1.7 |
| Agranulocytosis | 57 | 1.6 |
| Pain, not elsewhere classified | 49 | 1.4 |
| Atrial fibrillation and flutter | 46 | 1.3 |
| Dehydration | 41 | 1.1 |
| Chronic obstructive pulmonary disease | 40 | 1.1 |
| Other diseases of digestive system | 39 | 1.1 |
| Cellulitis | 41 | 1.1 |
| Malignant neoplasm of pancreas | 35 | 1.0 |
| Cerebral infarction | 37 | 1.0 |
| Acute myocardial infarction | 32 | 0.9 |
| Pulmonary embolism | 34 | 0.9 |
| Pneumonitis | 31 | 0.9 |
| Other noninfective gastroenteritis and colitis | 32 | 0.9 |
| Chest or throat pain | 31 | 0.9 |
| Complications of genitourinary prosthetic devices, implants, and grafts | 34 | 0.9 |
| Other bacterial intestinal infections | 29 | 0.8 |
| Disorders of continuity of bone | 28 | 0.8 |
| Syncope and collapse | 29 | 0.8 |
| Malignant neoplasm of colon | 26 | 0.7 |
| Aplastic anemia | 24 | 0.7 |
| Non-insulin-dependent diabetes mellitus | 24 | 0.7 |
| Other disorders of fluid, electrolyte, and acid-base balance | 27 | 0.7 |
| Pleural effusion | 24 | 0.7 |
| Malignant neoplasms of liver and intrahepatic bile ducts | 21 | 0.6 |
| Diffuse non-Hodgkin’s lymphoma | 22 | 0.6 |
| Diverticulitis | 23 | 0.6 |
| Altered mental status | 23 | 0.6 |
| Malaise and fatigue | 20 | 0.6 |
| Intracranial injury | 20 | 0.6 |
| Femur fracture | 20 | 0.6 |
| Complications of procedures, not elsewhere classified | 20 | 0.6 |
